# Cytotoxic and apoptotic effects of heat killed *Mycobacterium indicus pranii* (MIP) on various human cancer cell lines

**DOI:** 10.1038/srep19833

**Published:** 2016-01-28

**Authors:** Menaga Subramaniam, Lionel L A In, Ashutosh Kumar, Niyaz Ahmed, Noor Hasima Nagoor

**Affiliations:** 1 Institute of Biological Science (Genetics and Molecular Biology), Faculty of Science, University of Malaya, 50603, Kuala Lumpur, Malaysia; 2 Department of Biotechnology, Faculty of Applied Sciences, UCSI University, 56000, Kuala Lumpur, Malaysia; 3 Pathogen Biology Laboratory, Department of Biotechnology, School of Life Sciences, University of Hyderabad, Professor C.R. Rao Road, Hyderabad, Andhra Pradesh 500046, India; 4 Centre for Research in Biotechnology for Agriculture (CEBAR), University of Malaya, 50603 Kuala Lumpur, Malaysia

## Abstract

*Mycobacterium indicus pranii* (MIP) is a non-pathogenic mycobacterium, which has been tested on several cancer types like lung and bladder where tumour regression and complete recovery was observed. In discovering the potential cytotoxic elements, a preliminary test was carried out using four different fractions consisting of live bacteria, culture supernatant, heat killed bacteria and heat killed culture supernatant of MIP against two human cancer cells A549 and CaSki by 3-(4,5-dimethyl thiazol)-2,5-diphenyl tetrazolium bromide (MTT) assay. Apoptosis was investigated in MCF-7 and ORL-115 cancer cells by poly-(ADP-ribose) polymerase (PARP) and DNA fragmentation assays. Among four MIP fractions, only heat killed MIP fraction (HKB) showed significant cytotoxicity in various cancer cells with inhibitory concentration, IC_50_ in the range 5.6–35.0 μl/(1.0 × 10^6^ MIP cells/ml), while cytotoxicity effects were not observed in the remaining fractions. HKB did not show cytotoxic effects on non-cancerous cells contrary to cancerous cells, suggesting its safe usage and ability to differentially recognize between these cells. Evaluation on PARP assay further suggested that cytotoxicity in cancer cells were potentially induced via caspase-mediated apoptosis. The cytotoxic and apoptotic effects of MIP HKB have indicated that this fraction can be a good candidate to further identify effective anti-cancer agents.

The use of bacteria in cancer treatment is a well-known approach which was championed by W. Coley and German physicians W. Busch and F. Fehleisen who reported recovery of neck and other cancers following an infection with *Streptococcus pyogenes*^1^. Following these discoveries, several other bacterial species have been found to elicit significant anti-tumour activity in both *in vitro* and *in vivo* systems such as Lactobacillus species on bladder cancer^2^ and attenuated Salmonella species in murine tumour models^3^. Mycobacteria may be yet another promising species as it has shown a long successful history in treating cancer, for instance, the bacillus Calmette-Gue’rin (BCG) vaccine derived from *Mycobacterium bovis* was reported to be effective in treating human bladder cancer^4^.

Bacteria based anti-tumour therapy possess several advantages over chemical based drug. Firstly, some bacteria are able to selectively replicate and accumulate within tumour due to hypoxia environment and inhibits tumour growth. Next, motile bacteria are able to spread throughout the tumour and help in targeting systemic diseases. They can readily express multiple therapeutic transgenes such as cytokines and pro-drug converting enzymes to eradicate tumour mass^5^.

*Mycobacterium indicus pranii* (MIP) or conventionally known as *Mycobacterium w* (M.w) is a non-pathogenic, cultivable Mycobacterium species; which is now used widely as a vaccine for a number of diseases^6^. This vaccine works by boosting up the patient’s immunity through the induction of CD4^+^ T helper 1 (Th-1) cells response to release cytokines IL-2, IL-12, IL-15 and IFN-γ in order to promote cell-mediated immunity, and has been reported to be safe for human use in the treatment of leprosy^6^, tuberculosis^7^, HIV infection^8^ and lung cancer^9^ diseases.

Apart from inducing the immune system, certain mycobacteria species were also reported to induce a direct cytotoxic effect on cancer cells. As reported by Saitoh and Morales, BCG and BCG components^4^ could induce cancer cell apoptosis, while *M. phlei* or mycobacterial cell wall and DNA components possess certain anti-tumour activity^10^. These findings provides an insight on exploiting MIP and its cellular components as a potential anti-cancer agent against various human cancer cell lines.

As such to date, only certain types of cancers were reported to show cytotoxic effects upon MIP treatment in *in vivo*^11^,^12^. Therefore, there is a great interest to discover MIP cytotoxicity on various human cancer cell lines to broaden its utility. In this study, we evaluated *in vitro* cytotoxicity effect of four different MIP fractions consisting of live bacteria, culture supernatant, heat killed bacteria and heat killed culture supernatant against various human cancer type namely breast, cervical, oral, lung, bladder, liver and prostate.

## Results

### Cytotoxic screening for active MIP fractions

**Figure 1:**
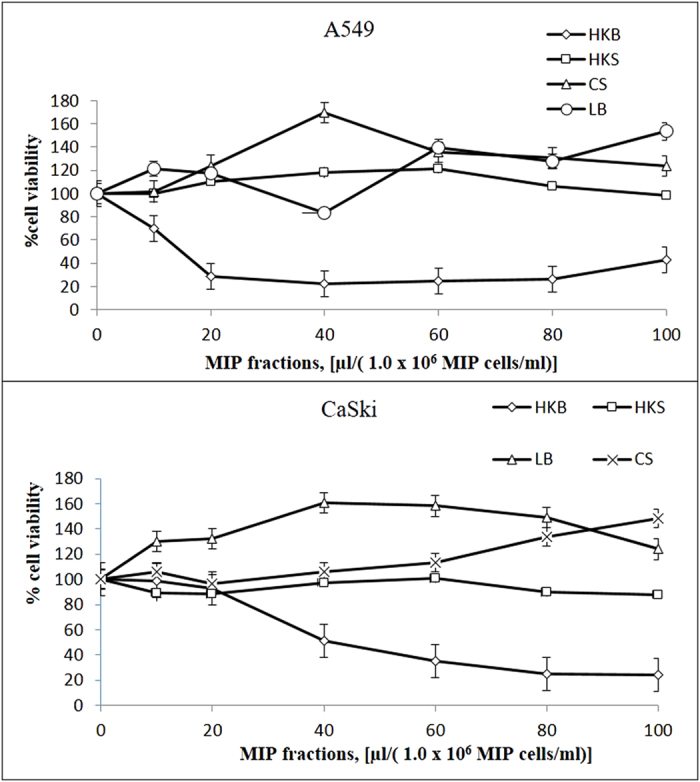
Cytotoxicity assay using MIP fractions at 24 hrs in human cervical carcinoma cell line (CaSki) and human lung carcinoma cell line (A549). MIP fractions: MIP live bacteria (LB), MIP culture supernatant (CS), MIP heat killed bacteria (HKB) and MIP heat killed culture supernatant (HKS). All MTT data were represented as mean ± SD of three independent experiments.

MIP was separated into four fractions: live bacteria (LB), culture supernatant (CS), heat killed bacteria (HKB) and heat killed culture supernatant (HKS). In identifying the active MIP fraction with cytotoxic effects, all four fractions were treated in two different cancer cell lines; cervical (CaSki) and lung (A549). The cell viability upon 24 hrs treatment was measured using MTT assay based on the mitochondrial activity in viable cells. In both cancer cells, only MIP HKB fraction showed cytotoxic effects where cell viability reduced to 24% in CaSki and 26% in A549, while the remaining fractions did not show any killing effects (Fig. 1). Thus, MIP HKB fraction was used here after to assess its cytotoxic consistency on various other cancer cell lines.

### *In vitro* cytotoxic effects of MIP HKB

**Figure 2:**
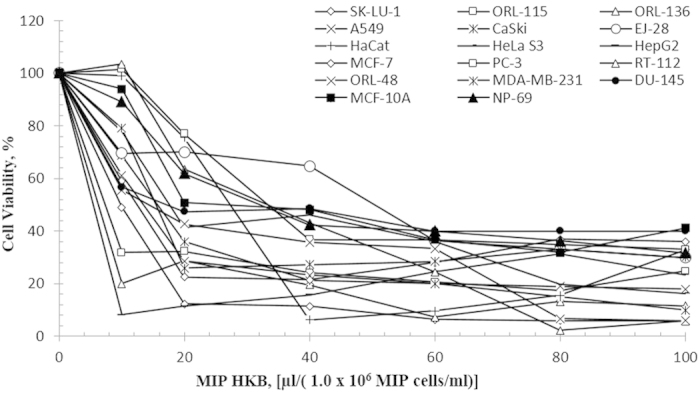
Cytotoxicity of MIP heat killed bacteria at 24 hrs in various human cancer cell lines by MTT assay. Bladder cancer cell lines (RT-112 and EJ-28); breast cancer cell lines (MDA-MB-231 and MCF-7); liver carcinoma cell line (HepG2); prostate cancer cell lines (PC-3 and DU-145); cervical carcinoma cell lines (CaSki, and HeLa S3); lung carcinoma cell lines (A549 and SK-LU-1); oral cancer cell lines (ORL-48, ORL-115 and ORL-136); immortalized human keratinocyte cell line (HaCaT); normal human nasopharyngeal epithelial cell line (NP-69); normal human breast epithelial (MCF-10A). Data is shown as mean ± S.D. of three independent replicates.

Cytotoxic applicability of the MIP HKB fraction was analyzed on various human cancer cell types (breast, cervical, lung adenocarcinoma, prostate, liver, bladder and oral) and non-cancerous cells (immortalized human cell lines from keratinocyte (HaCaT), nasopharyngeal epithelial (NP-69) and breast epithelial (MCF-10A)) as controls at 24 hrs treatment using MTT cell viability assay. Results indicated that cells treated with MIP HKB induced cytotoxicity in a dose dependent manner, similar to that of CaSki and A549 cells (Fig. 2). Table 1 shows IC_50_ values of MIP heat killed bacteria on various human cancer cell lines. Highest cytotoxicity was observed in liver cancer cell, HepG2 with an IC_50_ of 5.6 μl/(1.0 × 10^6^ MIP cells/ml) at 24 hrs. Oral cancer cells showed the second highest cytotoxicity (ORL-48, ORL-115 and ORL-136), with IC_50_ values of 13.6 μl/(1.0 × 10^6^ MIP cells/ml), 7.8 μl/(1.0 × 10^6^ MIP cells/ml) and 5.9 μl/(1.0 × 10^6^ MIP cells/ml), respectively followed by lung and breast cancers. HaCaT, MCF-10A and NP-69 with IC_50_ values of 23.5 μl/(10^6^ cells/ml), 25.7 μl/(10^6^ cells/ml) and 32.9 μl/(10^6^ cells/ml) respectively, implies that concentrations higher than these values are toxic to non-cancerous cells. Also IC_50_ values higher than 23 μl/(1.0 × 10^6^ MIP cells/ml) in several cancer cells (PC-3, 34.5 μl/(1.0 × 10^6^ MIP cells/ml); EJ-28, 51.9 μl/(1.0 × 10^6^ MIP cells/ml); RT-112, 35.5 μl/(1.0 × 10^6^ MIP cells/ml) indicates heat killed MIP treatment is less effective in these cancer cells.

### DNA fragmentation and PARP assay

**Figure 3:**
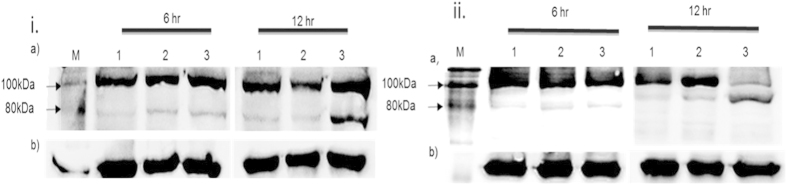
Effects of MIP HKB on PARP cleavage at 6 and 12 hr. i. MCF-7 cell line; ii. ORL-115 cell line. **(a)** Cells were treated with MIP HKB at 6 and 12 hrs and PARP was measured by the Western blot analysis. **(b)** GAPDH was used as a loading control. Lane M: Biotinylated protein ladder; Lane 1: Untreated cells; Lane 2: PBS treated cells; Lane 3: HKB treated cells.

To further analyze the mode of cell death upon MIP HKB treatment, MCF-7 and ORL-115 cells were selected as model cell lines owing to it having an IC_50_ value below the HaCat cell line threshold. The morphological changes in both cells shows MIP induced apoptotic cell death (data not shown). The PARP cleavage assay was carried out to validate the apoptosis mediated cell death in both cell lines. Results showed cleavage of the inhibitory fragment from the 116 kDa full length PARP into an 89 kDa fragment (Fig. 3). Cells were treated with PBS and MIP HKB, 12 μl/(1.0 × 10^6^ MIP cells/ml) for MCF-7 while 7.8 μl/(1.0 × 10^6^ MIP cells/ml) for ORL-115 in a time dependent manner at 6 and 12 hrs to observe the initiation and progression of apoptosis. The housekeeping gene, GAPDH was used as a protein normalization and loading control.

**Figure 4:**
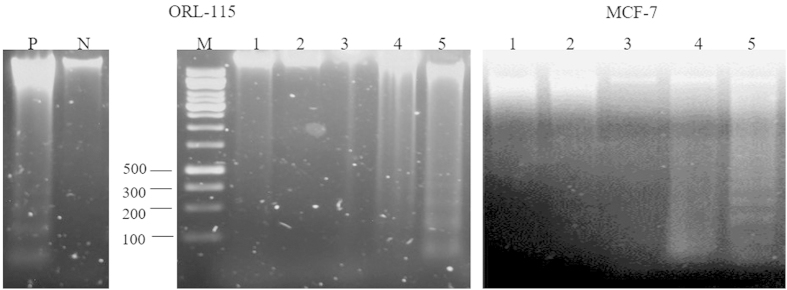
DNA gel electrophoresis of inter-nucleosome DNA fragmentation in 1.5% (w/v) agarose gel at 6, 12 and 24 hrs treatment in MCF-7 and ORL-115 cell lines. Lane P: positive control with 1’-(S)-1’-acetoxychavicol acetate (ACA) treated MCF-7 cells^19^; Lane N: negative control (untreated MCF-7 cells); Lane M: DNA molecular weight marker; Lane 1: Untreated cells; Lane 2: PBS treated cells for 24 hrs; Lane 3: HKB treated at 6 hrs; Lane 4: HKB treated at 12 hrs; Lane 5: HKB treated at 24 hrs. DNA laddering was demonstrated in cells treated with MIP HKB in Lane 5.

DNA fragmentation assay was carried out to confirm and observe the occurrence of late apoptosis in MCF-7 and ORL-115 cells at 6, 12 and 24 hrs. A 150 bp to 200 bp laddering of DNA at 12 hrs upon MIP exposure in MCF-7 indicates a strong hallmark of late apoptotic events (Fig. 4). Ladder formation was absent in both untreated and PBS treated cells, which showed that the appearance of apoptotic DNA fragments were due to the cytotoxic effect of MIP HKB treatment.

## Discussion

In cancer treatment, MIP is used as an adjuvant to radiation therapy in patients with bladder cancer^12^ and to chemotherapy plus radiotherapy in non-small cell lung cancers^10^. Four types of fractions can be obtained from MIP: LB, HKB, CS and HKS, with the most widely used fractions being the HKB fraction^13^,^14^ and CS fraction^15^. While past studies have cited autoclaving for 20 mins at 15 lb/in^2^ as the most common heat killing method, this method may denature important and biologically active proteins, which led us to heat-kill MIP at 60 °C, which was also found to be sufficient in killing MIP cultures. When all MIP fractions were cultivated in 7H10 agar, no growth was observed after a week of incubation, with the exception of LB fraction, thus confirming the complete killing of MIP at 60 °C. This method is recommended because even though MIP cultures were completely heat-killed, other intracellular and extracellular proteins/precursors potentially responsible for its cytotoxicity would likely remain intact.

MIP HKB demonstrated therapeutic cytotoxicity against most of the tested human cancer cells, and was less potent towards non-cancerous human cells based on its high IC_50_ value. The difference in MIP selectivity between non-cancerous cells and cancer cells may be due to differences in growth rate, which results from the presence of distinct cell surface receptors, differences in the uptake of certain drugs and the method used for assessment of toxicity^16^. This selective cytotoxic effect is an important criteria to ensure the drug’s safety and efficacy in patients with minimal side effects. Experimental results imply MIP HKB selectivity in MDA-MB-231, MCF-7, CaSki, A549, SK-LU-1, DU-145, HepG2, ORL-48, ORL-115 and ORL-136 with IC_50_ values between 5.6 to 21 μl/(1.0 × 10^6^ MIP cells/ml), all of which are lower than non-cancerous cells. According to a previous study on apoptotic cell death in *in vitro* by Pandey *et al.*, 2011^15^, 60–70 μl of MIP is required to induce cell death in 40–45% mouse peritoneal macrophages while in this study, 60–70 μl/(1.0 × 10^6^ MIP cells/ml) of MIP HKB induced 75% cell death. This clearly shows a tremendous reduction in MIP dose when a 60 °C heat kill technique was applied compared to autoclave heat kill technique.

This study also identified that cancer cell death was induced via apoptosis in MCF-7 and ORL-115 cells as confirmed through PARP and DNA fragmentation assays. Apoptosis is a cell suicide mechanism to remove redundant, damaged, or infected cells through a group of caspases activation. These caspases are grouped into initiator (caspases-2, -8, -9, and -10) and effector (caspases-3, -6 and -7) caspases. Effector caspases are responsible for dismantling of necessary cell components, which results in morphological and biochemical changes that characterize apoptotic cell death as cytoskeletal rearrangement, cell membrane blebbing, nuclear condensation and DNA fragmentation. The DNA fragmentation observed in MCF-7 cells, a caspase-3 deficient cell line, was most probably due to other effector caspases, such as, caspase 7 activation^17^.

## Materials and Methods

### Materials

Dulbecco modified Eagle medium (DMEM) supplemented with 4.5 g glucose/L and 300 mg/L L −glutamine was purchased from Hyclone Laboratories Inc, Logan, Utah. Roswell Park Memorial Institute 1640 (RPMI-1640) was purchased from Thermo Scientific Hyclone, USA. Fetal bovine serum (FBS) was purchased from Lonza Inc. (Allendale, New Jersey, USA). Keratinocyte serum-free medium (KSFM) and TRIzol reagent were obtained from Invitrogen, Grand Island, New York. Minimum essential medium alpha (MEM-α) was purchased from Life Technologies, USA. Cisplatin and 3–(4, 5-dimethylthiazol-2-gl)-2, 5-diphenyl-tetrazoliumbromide (MTT) reagents were obtained from EMD Chemicals Inc. Middlebrook 7H10 agar and 7H9 broth were obtained from Sigma-Aldrich, Germany. *Mycobacterium indicus pranii* was provided by Prof. Dr. Niyaz Ahmed, Department of Biotechnology & Bioinformatics, School of Life Sciences, University Hyderabad, India.

### Cultivation of cancer cells

A total of fourteen human cancer and three non-cancerous cell lines were used in this study: breast adenocarcinoma cell lines (MCF-7 and MDA-MB-231), hepatocyte carcinoma cell line (HepG2), cervical cancer cell lines (CaSki and HeLa S3), prostate carcinoma cell lines (PC-3 and DU-145), lung adenocarcinoma cell lines (A549 and SK-LU-1), oral cancer cell lines (ORL-48, ORL-115 and ORL-136) and bladder cancer cell lines (RT-112 and EJ-28). Immortalized human cell lines from nasopharyngeal epithelial (NP-69), breast epithelial (MCF-10A) and keratinocyte (HaCaT) were used as representatives of non-cancerous cells. All cell lines were obtained from ATCC except human oral cancer cell lines which were obtained from Cancer Research Initiative Foundation (CARIF, Malaysia) and NP-69 is a gift from Prof GSW Tsao, The University of Hong Kong^18^. HaCaT, HepG2, CaSki and HeLa S3 were cultured in DMEM, while MCF-7, PC-3, MDA-MB-231, A549, RT-112, EJ-28 and DU-145 cells were cultured in RPMI 1640, supplemented with 10.0% (v/v) FBS. SK-LU-1 cells were cultured in MEM-α supplemented with 10% (v/v) heat inactivated FBS. NP-69 cells were cultured in keratinocyte serum-free medium (KSFM) (Gibco, USA) supplemented with 1 × 2.5 μg human recombinant epidermal growth factor (rEGF) (Gibco, USA) and 1 × 2.5 mg bovine pituitary extract (Gibco, USA). The MCF-10A was grown in serum-free mammary epithelial basal media (MEBM, Lonza, USA) supplemented with cholera toxin (100 ng/ml). All cells were grown as monolayers and were maintained in a humidified CO_2_ incubator at 37 °C in 5.0% CO_2_ and 95.0% air. Each cell line was seeded at a density of 1.0 × 10^4^ cells/well in 96-well plates (100 μl/well) and left overnight in the incubator prior to commencement of treatment.

### Bacterial cultures

MIP was cultured in Middlebrook (MB) 7H9 broth supplemented with 5ml glycerol, 0.2% Tween-80, 10% albumin-dextrose complex enrichment (ADC) and incubated at 37 ^o^C, 100 rpm agitation until 1.5 OD_600_.

### Preparation of MIP fractions

Original MIP suspension containing 6.0 × 10^9^ MIP cells/ml at 1.5 OD_600_ as determined using CFU plate count assays was harvested to prepare MIP fractions: live bacteria (LB), culture supernatant (CS), heat killed bacteria (HKB) and heat killed culture supernatant (HKS). The suspension was centrifuged at 3500 rpm for 15 mins and supernatant (CS fraction) and MIP pellets were separated. Pellets were suspended in 0.9% (w/v) sodium chloride and 0.01% (w/v) thimerosal and centrifuged at 3500 rpm for 10 mins. The supernatant was then discarded. The pellet containing MIP cells was washed and resuspended in the original volume in sterile PBS, creating the LB fraction. HKB and HKS fractions were then prepared by heat inactivating the CS and LB fractions at 60 °C for 20 mins in a water bath. Various cell lines were treated with different volumes [10.0 to 100.0 μl/(1.0 × 10^6^ MIP cells/ml)] of MIP fractions.

### MTT cell viability assay

MTT assay was carried out to measure cytotoxic effects of MIP fractions on various cancer cell lines. 3-(4, 5-dimethylthiazol-2-yl)-2, 5-diphenyltetrazolium bromide (MTT) is a substrate which is reduced by dehydrogenase enzymes present in the mitochondria of viable cells. In MTT assay, the intensity of the purple formazan product was measured and used to quantify viable cells in culture. A total of 100.0 μL of cells were plated per well (1.0 × 10^4^ cells/well), incubated overnight, and treated with MIP fractions at various concentrations then incubated for 24 hrs. 20 μL of MTT reagent (5.0 mg/mL) was added to each well. The plate was left on a shaker for 10 mins and incubated in the dark at 37 °C. After 1 hr of incubation, the spent medium containing excess dye was aspirated and 200 μL of DMSO added to dissolve the purple formazan precipitates. Results were obtained using micro-titer plate reader (Tecan Sunrise, Switzerland), to detect absorbance at a test wavelength of 570 nm, and a reference wavelength of 650 nm. From absorbance data obtained, a graph was plotted employing the following equation: Viability (%) = [100% – cytotoxicity (%)]; where cytotoxicity (%) = [(absorbance value of solvent - absorbance value of MIP fraction)/absorbance value of untreated cells] × 100%. IC_50_ values for MIP HKB fraction were determined from the graph at 50% cell viability.

### PARP cleavage assay

The occurrence of apoptosis was assessed based on the proteolytic cleavage of PARP by caspase-3. Briefly, 2.0 × 10^6^ cells/mL were treated with MIP HKB (IC_50_) and total proteins were extracted using the NE-PERW nuclear and cytoplasmic extraction kit according to manufacturer’s protocol. Fractionation was done using SDS-PAGE and electro-transferred onto nitrocellulose membranes. All membranes were blocked with 5% w/v BSA, 1 × TBS, 0.1% Tween-20 at room temperature with gentle shaking for 90 mins, and incubated with primary antibodies: GAPDH (1:1000) and PARP (1:1000) overnight at 4 °C, followed by detection using HRP-conjugated secondary antibodies (Cell Signaling, USA), and Super Signal West Pico chemiluminescent substrate. Images were captured using the Fusion FX7 imaging system (Vilber Lourmat, France). Apoptosis was represented by cleavage of 116 kDa full length PARP into an 89 kDa product.

### DNA fragmentation assay

Cells were treated with 1x PBS and MIP HKB at 6, 12 and 24 hrs before harvesting, and total DNA was extracted from both untreated and treated cells using the Suicide Track^TM^ DNA Ladder isolation kit according to the manufacturer’s protocol. MCF-7 cells treated with the apoptosis inducing agent, 1’-(S)-1’S-1’-acetoxychavicol acetate (ACA) served as a positive control. Extracted DNA was analyzed on a 1.5% (w/v) agarose gel electrophoresis and stained with ethidium bromide. Fragmentation of DNA was observed under UV illumination and visualized using a gel documentation system (Alpha Inotech, USA).

## Conclusion

Currently *Mycobacterium indicus pranii* (MIP) has only been tested on lung and bladder cancers with tumour regression and complete recovery observed. Its effects on other cancer cell types have yet to be determined. The MIP HKB fraction was identified as the most potent cytotoxic fraction compare to LB, CS and HKS in terms of its low IC_50_ values and induction of apoptotic cell death in breast and oral cancer cells. Therefore the cytotoxic and apoptotic effects of MIP HKB in these two human cancer cells indicate that it can be a good candidate for further pharmacological studies to identify effective biologically active anti-cancer agents. In summary, this study has proven that MIP HKB, killed at 60 °C can inhibit the growth of various human cancer cell lines through activation of apoptosis.

## Additional Information

**How to cite this article**: Subramaniam, M. *et al.* Cytotoxic and apoptotic effects of heat killed *Mycobacterium indicus pranii* (MIP) on various human cancer cell lines. *Sci. Rep.* **6**, 19833; doi: 10.1038/srep19833 (2016).

## Figures and Tables

**Table 1:** IC_50_ values of MIP HKB treated fraction on various human cancer and non-cancerous cell lines.

Cell type	Cell line	IC_50_ [μl/(1.0 × 10^6^MIP cells/ml)]
Immortalized human keratinocytes	HaCat	23.5 ± 5.4
Immortalized human nasopharyngeal epithelial	NP-69	32.9 ± 1.0
Immortalized human breast epithelial	MCF-10A	25.7 ± 0.6
Breast cancer	MDA-MB-231	15.4 ± 0.1
	MCF-7	12.0 ± 0.7
Cervical cancer	CaSki	15.9 ± 1.8
	HeLa S3	21.1 ± 2.2
Lung cancer	A549	14.3 ± 1.3
	SK-LU-1	7.8 ± 2.8
Prostate cancer	PC-3	34.5 ± 1.6
	DU-145	18.4 ± 1.7
Liver cancer	HepG2	5.6 ± 0.2
Bladder cancer	EJ-28	51.9 ± 2.0
	RT-112	35.5 ± 3.2
Oral cancer	ORL-48	13.6 ± 1.0
	ORL-115	7.8 ± 1.0
	ORL-136	5.9 ± 0.5
